# Two cases of mimics of bone metastasis in breast cancer

**DOI:** 10.1259/bjrcr.20170091

**Published:** 2017-12-16

**Authors:** Aung Win Tin, John Hardman, Geoffrey Naisby

**Affiliations:** 1 Department of Radiotherapy and Oncology, James Cook University Hospital, Middlesbrough, UK; 2 Department of Radiology, James Cook University Hospital, Middlesbrough, UK

## Abstract

In patients with breast cancer, the appearance of sclerotic bone lesions on imaging should raise the suspicion of skeletal metastases. However, before making the diagnosis it is important to consider the clinical context and remember that there are conditions that can mimic bone metastasis. We present two cases of mimics of bone metastasis: systemic mastocytosis and osteopoikilosis. These cases demonstrate clinical and radiological characteristics that would make a diagnosis of bone metastasis less likely, and highlight the need for an awareness of mimics of bone metastasis.

## Background

Bone is the commonest site of metastasis in breast cancer.^1^ However, the appearance of widespread bony sclerosis cannot, in isolation, conclusively lead to a diagnosis of skeletal metastasis. Here we present two cases of very rare conditions that mimic skeletal metastasis.

***Mastocytosis ***is characterized by an abnormal proliferation of mast cells. It affects approximately two cases per 100,000/year.^2^ It has been classified into two groups: cutaneous mastocytosis (CM); confined to the skin, and systemic mastocytosis (SM) which is a clonal and disseminated condition.^3^

***Osteopoikilosis*** is an asymptomatic osteosclerotic dysplasia with an estimated incidence of one in 50,000. Osteopoikilosis has variable onset and can occur sporadically or as an inherited disorder.^4^

## Case 1

A 57-year-old female was referred to Oncology for consideration of adjuvant treatment following right breast wide local excision with sentinel lymph node biopsy in August 2011 for Grade 2, lymph node-negative, ER-positive and HER2-negative invasive ductal carcinoma. She stated that she had been treated for a skin condition called “urticaria pigmentosa” (UP) many years earlier. She went on to have adjuvant radiotherapy to the right breast and hormonal therapy.

**Figure 1.**
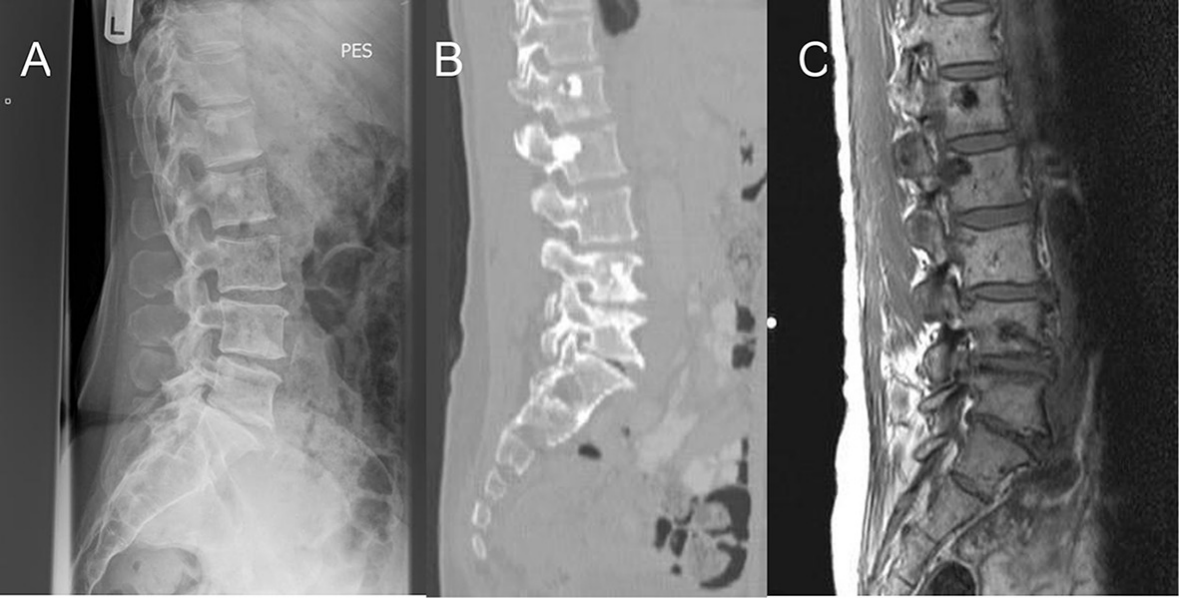
Additional imaging requested to investigate increased density in the lumbar spine on DEXA scan of a 57-year-old female with early-stage breast cancer (Case 1). (a) X-ray of lumbosacral spine showing focal bony sclerosis at T12 and L1. (b) Sagittal section of CT of lumbosacral spine showing sclerotic lesions in L1 to L4. (c) Sagittal *T*_1_ section of MRI of lumbosacral spine showing corresponding low signal lesions in the vertebral bodies.

**Figure 2.**
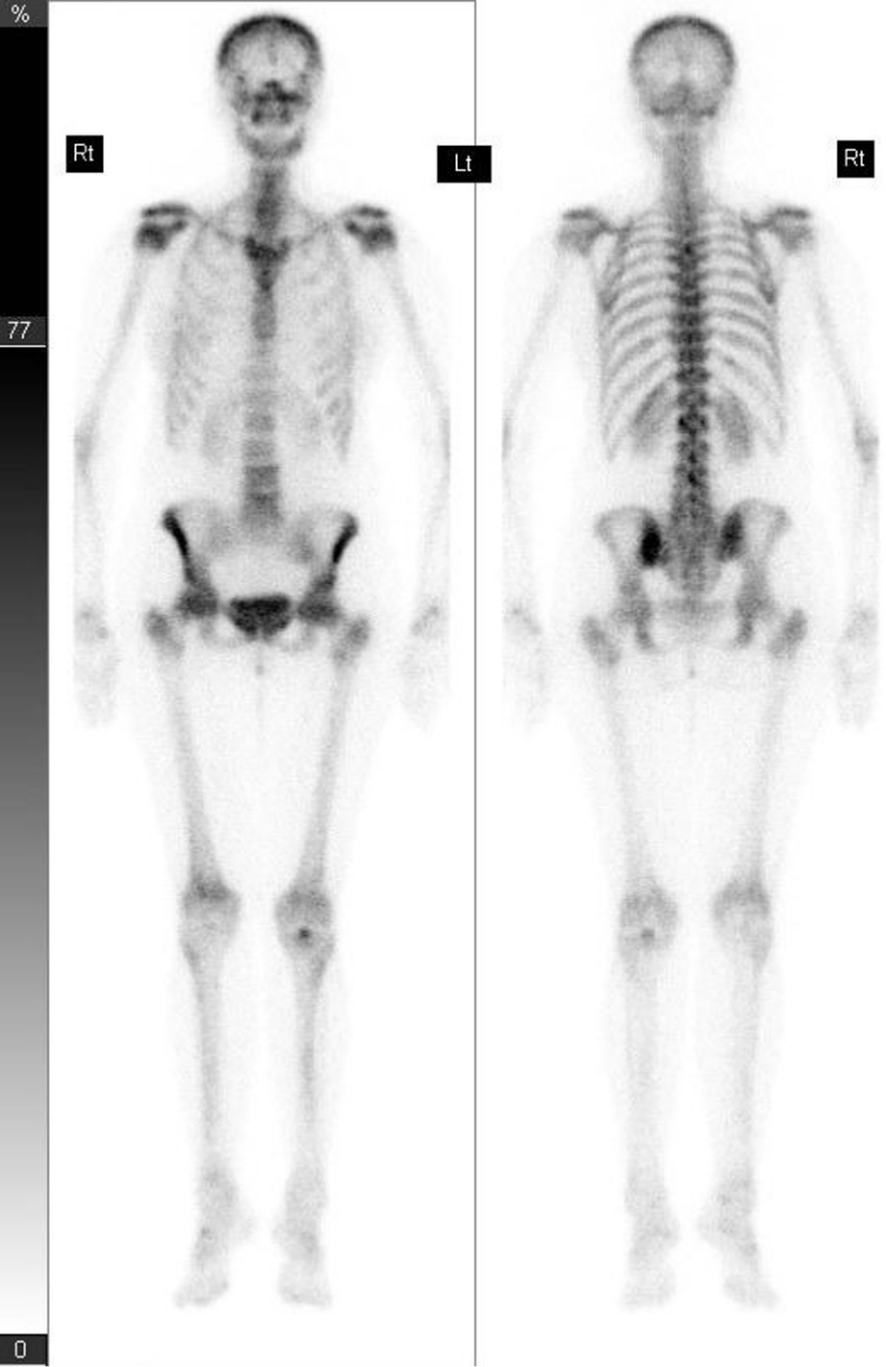
Isotope bone scan for a 57-year-old female with early stage breast cancer (Case 1) showing subtle focal uptake in L1, possibly L3, left sacroiliac joint, left iliac bone and left acetabulum. There was surprisingly little uptake considering the X-ray appearances. Potential explanations provided for the relative lack of uptake on the bone scan were: inactive metastatic disease, perhaps in response to Letrozole; or an aggressive osteoclastic component masking uptake.

The patient’s baseline DEXA scan revealed increased density in the lumbar spine, which prompted further investigations including X-ray of the lumbosacral spine and CT scan of the chest, abdomen and pelvis (Figure 1). These showed multiple bony sclerotic foci in the thoracolumbar spine and acetabulum consistent with metastatic bone disease. No soft tissue changes were noted on CT. The patient also had an isotope bone scan (Figure 2) which showed only subtle focal uptake in the lumbar spine, left sacroiliac joint, left iliac bone and left acetabulum, that was discordant with plain films. At the time the potential explanations provided for the relative lack of uptake on the bone scan were: inactive metastatic disease, perhaps in response to Letrozole; or an aggressive osteoclastic component masking uptake.

Following these imaging results, it was explained to the patient that it was unusual for the X-ray appearances to be more striking than the bone scan abnormalities but that she did indeed have skeletal metastases. Therefore she was started on monthly Zoledronic acid. Her CA-15.3 and bone profile were in normal limits.

A repeat CT scan in 2013 and MRI scan of the spine in 2014 (Figure 1) showed appearances consistent with skeletal metastases. The MRI scan showed no new spinal lesions. Meanwhile the patient remained extremely well with no bone pain. At a recent consultation, she recalled having had multiple investigations in the 1990s, culminating in a bone marrow aspiration. These investigations occurred approximately 250 miles away from her current hospital.

Following this, two dermatology letters from 1993 were obtained and detailed a history of worsening skin problems and described diffuse pigmented patches distributed proximally over her body, typical of UP. Investigations including bone marrow aspirate, abdominal ultrasound scan and skeletal survey, to ensure that disease was limited to the skin, were requested. The results of these investigations were unremarkable, except the bone marrow aspirate which showed mast cells in the bone marrow, suggesting SM. Subsequently, she was commenced on twice-weekly phototherapy (PUVA) and informed that her “bones had small holes in them”.

In the context of SM, her imaging was re-examined by the radiology team. It is now considered that given the lack of uptake on the bone scan and the static nature of the bone lesions between the CT scan in 2013, and MRI scan in 2014, that these radiological abnormalities are more consistent with SM than inactive metastatic breast cancer. The fact that she had otherwise early-stage ER-positive, HER2-negative breast cancer reduces the likelihood of having bone metastasis at presentation. The oncologist explained the revised opinion to the patient and apologized for any anxiety caused regarding her prognosis. She continued on adjuvant letrozole and was referred to a rheumatologist for follow-up for SM.

## Case 2

A 50-year-old female was referred to oncology for consideration of adjuvant treatment following a left-sided mastectomy and axillary clearance in 2012. She was diagnosed with a Grade 2, lymph node-positive, ER-positive, HER2-negative invasive lobular carcinoma. Her CA-15.3 and bone profile were within normal limits.

**Figure 3.**
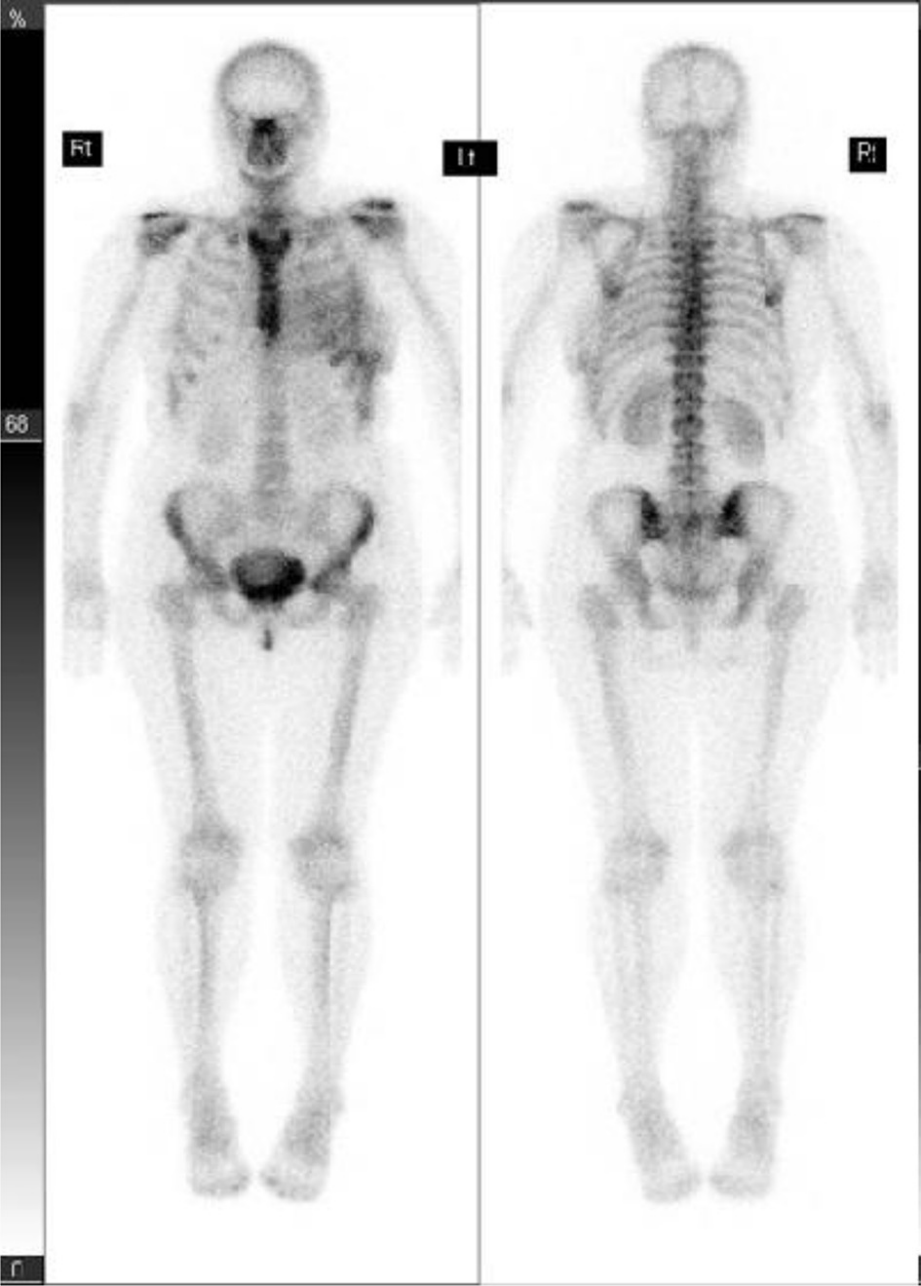
Isotope bone scan of a 50-year-old female with node-positive breast cancer (Case 2) showing subtle increased uptake in the left anterior chest consistent with recent breast surgery but no evidence of bone metastases.

**Figure 4.**
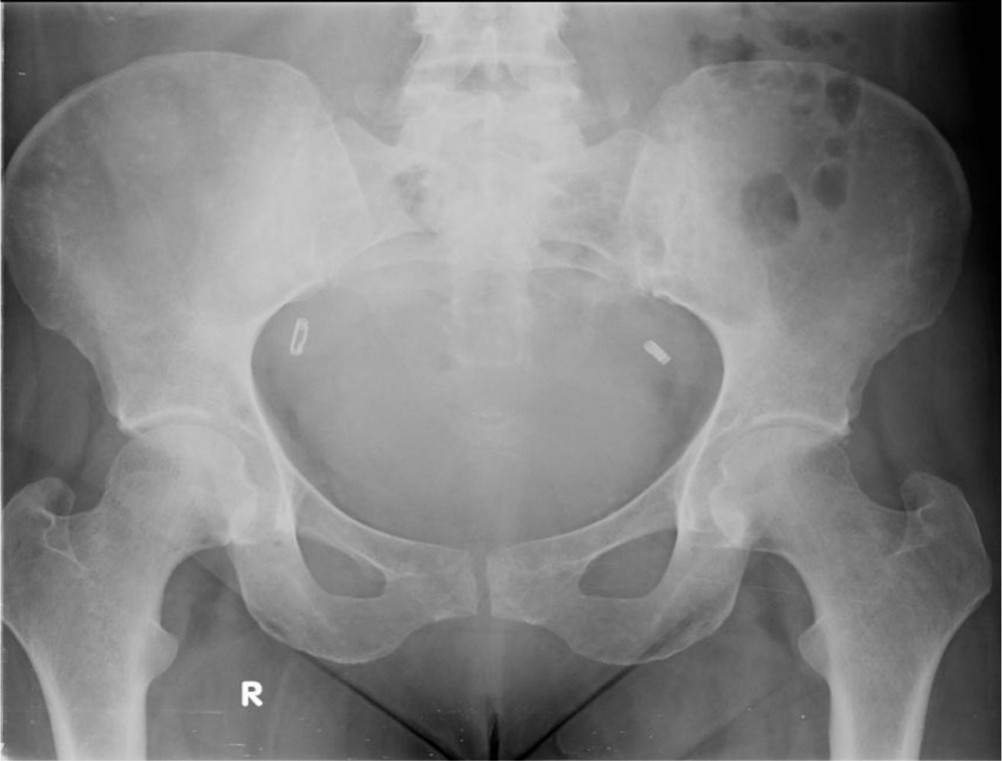
X-ray of pelvis of a 50-year-old female with node-positive breast cancer (Case 2) showing multiple sclerotic lesions in the iliac wings and proximal femora.

**Figure 5.**
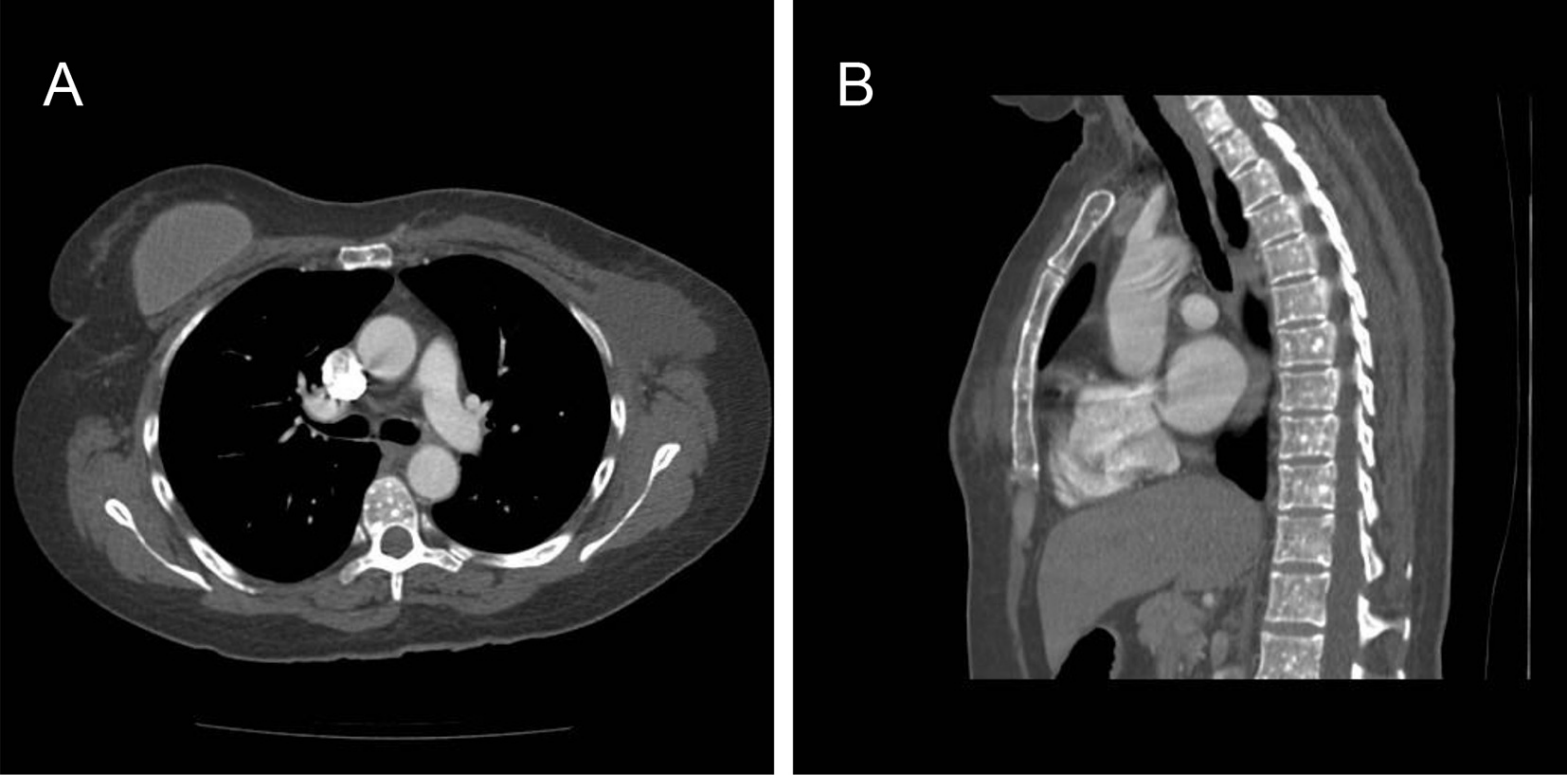
CT scan of a 50-year-old female with node-positive breast cancer (Case 2). (a) Axial CT image of the thorax showing multiple, small, rounded sclerotic foci in the T7 vertebral body. (b) Sagittal CT image showing multiple, small, rounded, sclerotic lesions throughout the vertebra and sternum.

The patient’s isotope bone scan showed no evidence of metastatic disease (Figure 3) but an X-ray of the pelvis showed sclerotic lesions mostly localized in the iliac wings (Figure 4), and CT scan of the thorax and abdomen showed multiple small sclerotic nodules throughout the entire skeleton (Figure 5). In the clinical context, the radiologist suggested that these lesions likely represented metastatic deposits, but that a benign cause such as osteopoikilosis should be considered.

The Breast MDT opinion was that given the normal bone scan, CA-15.3, bone profile and absence of bone-related symptoms, a diagnosis of bone metastasis seemed unlikely. Therefore it was explained to the patient that the bony abnormalities detected on X-ray and CT were most likely to be due to osteopoikilosis. She was subsequently treated with adjuvant chemotherapy followed by radiotherapy to the left chest and left supraclavicular fossa, alongside hormonal therapy. Three years later, there has been no evidence of disease recurrence. Further CT scans performed in 2013 and 2014 showed stable appearances in the bones.

## Discussion

### Systemic mastocytosis

Mast cells are normal cells in the body, usually localized beneath or in the epithelium close to vessels, nerves, smooth muscle cells and glandular tissue. But they do not circulate in peripheral blood.^5^ Granules within mast cells contain a broad range of chemicals, referred to as “mast cell mediators” which include: histamine, heparin and leukotriene C4 among others.^6^

The majority of patients with SM have a favourable prognosis. In particular those with indolent SM, as in Case 1, will have a near normal life-expectancy.^7^ In contrast, those with aggressive SM and mast cell leukaemia have a median survival of under 2 years.^8^

The symptoms of SM may be categorized as: skin symptoms relating to mast cell “release” and those caused by non-cutaneous organ infiltration. Skin manifestations are the most common, particularly UP, which occurs in 90% of patients with SM.^2^ Symptoms caused by non-cutaneous manifestations of SM will most frequently affect the bone marrow, gastrointestinal tract, lymph nodes, liver, spleen and urogenital system. Approximately 70% of patients with SM will have skeletal involvement.^9^ They may be asymptomatic or experience arthralgia, bone pain or pathological fractures from secondary osteoporosis. Bone sclerosis is present in 3–8% of patients. The characteristic radiological findings of SM-related bone disease are the presence of multiple foci of sclerosis, alternating with zones of apparently normal or reduced bone intensity.^10^

Bisphosphonate therapy should be started in patients with SM-induced osteoporosis or severe osteopenia (T score < –2). Bisphosphonates may also be considered as prophylactic treatment for patients with SM who have osteolyses and risk factors for osteoporosis.^11^ Therefore the commencement of zoledronic acid could be justified in Case 1, even though it was given mistakenly to reduce the risk of skeletal events from bone metastases.

### Osteopoikilosis

Osteopoikilosis is usually identified in cases where there has been an incidental finding of bony sclerotic lesions. However, it may also coexist with numerous developmental dysplasias.^12^ Radiologically, it is characterized by the presence of multiple, punctuate, sclerotic, rounded or oval, homogenous foci^4^ which often follow a symmetrical and periarticular distribution. The most commonly affected sites are the phalanges, carpal bones and metacarpals.^13^ Additionally, they usually appear inactive on isotope bone scans and fluoro-deoxyglucose positron emission tomography scans.^14^ These features distinguish osteopoikilosis from metastatic bone disease, which tends to be non-homogenous in appearance and rarely affects below the knee or elbow.

## Learning points

1. In patients with breast cancer, sclerotic bony lesions on imaging should raise the possibility of skeletal metastases. However, before making this diagnosis, it is important to consider potential mimics of bone metastases, including rare conditions such as SM and osteopoikilosis.
2. As demonstrated in both of these cases, the diagnosis of skeletal metastasis proved less likely given the context of inactive appearance on isotope bone scans, stable nature of the lesions on subsequent radiological investigation, normal tumour markers and absence of symptoms. Furthermore, in Case 1, a finding of skeletal metastasis conflicted with what was otherwise an early-stage ER-positive, HER2-negative breast cancer.
3. Other potential mimics include benign abnormalities such as fibrous dysplasia or osteopathia striata and malignancies such as lymphoma and myeloma.^15^
4. Making the correct diagnosis is critical to spare patients undue anxiety about their prognosis and avoid unnecessary treatments. Fortunately for the patient in Case 1, her additional treatment was with bone-protecting agents, which could be justified given the prophylactic role of bisphosphonates in SM-induced bone disease.^11^
5. There are multiple factors that could have enabled an earlier, correct diagnosis for the patient with SM: more effective communication to the patient from the dermatologist regarding the diagnosis and potential complications of SM; up-to-date patient held medical records detailing a complete past medical history and improved clinician awareness regarding possible mimics of sclerotic bony metastasis.

In conclusion, SM and osteopoikilosis are rare conditions that can be mistaken for bone metastasis. Please give them consideration.

## Consent

Written informed consent for the case to be published (including images, case history and data) was obtained from the patient(s) for publication of this case report, including accompanying images.
